# 

**Published:** 1885-03

**Authors:** 


### BOOKS RECEIVED.

---

BOOKS AND PAMPHLETS RECEIVED.

*Diphtheria, Croup, etc., or the Membranous Diseases*, by G. B. Galentin, M. D. J. H. Vail Co. Chicago: W. T. Keener.

*A Text-Book of Pathological Anatomy and Pathogenesis*, by Ernst Ziegler. Wm. Wood & Co. Chicago: W. T. Keener.

*On a New Method of Recording the Motions of the Soft Palate*, by Harrison Allen, M. D. P. Blakiston Son & Co. Chicago: W. T. Keener.

*Tex-Book of Medical Jurisprudence and Toxicology*, by John J. Reese, M. D. P. Blakiston, Son & Co. Chicago: W. T. Keener.

*A Manual of Diseases of the Throat.* Vol. II. By Morell McKenzie, M. D. Wm. Wood & Co. Chicago: W. T. Keener.

*Eleventh Annual Report of the State Board of Health of Michigan.*

*A System of Human Anatomy including its Medical and Surgical Relations.* Section vi. By Harrison Allen, M. D. Henry C. Lea's Son & Co.

*A Manual of Obstetrics*, by Edward L. Partridge, M. D. Wm. Wood & Co. Chicago: W. T. Keener.

*Practical Manual of Diseases of Women and Children*, by Eustace Smith, M. D. Wm. Wood & Co. Chicago: W. T. Keener.

*Practical Manual of Diseases of Women and Uterine Ther-*

*apeutics*, by H. Macnaughton Jones, M. D. D. Appleton & Co. Chicago: Jansen, McClurg & Co.

*Osteotomy and Osteoclasis for Deformities of the Lower Extremities*, by C. T. Poore, M. D. D. Appleton & Co. Chicago: Jansen, McClurg & Co.

*The Lock-Jaw of Infants*, by J. F. Hartigan, M. D. Birmingham & Co.

*A Practical Treatise on Fractures and Dislocations*, by Frank H. Hamilton. Henry C. Lea's Son & Co. Chicago: Jansen, McClurg & Co.

*The Ear: its Anatomy, Physiology and Diseases*, by Chas. H. Burnett, M. D. Henry C. Leas Son & Co. Chicago: Jansen, McClurg & Co.

*Atlas of Female Pelvic Anatomy*, by D. Berry Hart. D. Appleton & Co. Chicago: Jansen, McClurg & Co.

*The Elements of Pathology.* By Edward Rendfleisch. Translated by W. H. Murcur, M. D. P. Blakiston, Son & Co. Chicago: W. T. Keener.

*Index Catalogue of the Library of the Surgeon-General's Office, Vol. V.*

*Medical Rhymes.* By Hugo Erichsen, M. D. J. H. Chambers & Co.

*Myths in Medicine and Old Time Doctors.* By A. C. Yanat, M. D. G. P. Putnam's Sons.

*Malaria and Malarial Diseases.* By Geo. W. Sternberg. Wm. Wood & Co. Chicago: W. T. Keener.

*Diseases of the Nose.* By Clinton Wagner, M. D. Birmingham & Co.

*A Manual of Dermatology.* By A. R. Robinson, M. D. Birmingham & Co.

*Medical Diagnosis, a Manual of Clinical Methods.* By J. Graham Brown. Birmingham & Co.

*Lectures on Diseases of the Rectum.* By J. W. Wright, M. D. Birmingham & Co.

*Surgical Delusions and Follies.* By John B. Roberts, A. M., M. D. P. Blakiston, Son & Co.

*Transactions of the Indiana State Medical Society, 1884.*

*Surgery of the Urinary Organs.* By Sir Henry Thompson. P. Blakiston, Son & Co.

*A Theoretical and Practical Treatise on the Haemorrhoidal Disease.* By Wm. Bodenhamer, A. M., M. D. Wm. Wood & Co. Chicago: W. T. Keener.

*A Practical Treatment on Massage.* By Douglas Graham. Wm. Wood & Co. Chicago: W. T. Keener.

*A Practical Treatise on the Diseases of the Ear.* By D. B. St. John Roosa. Sixth Edition. Wm. Wood & Co. Chicago: W. T. Keener.

*Swayne's Obstetric Aphorisms.* Eighth Edition. P. Blakiston, Son & Co.

*Micro-Organisms and Disease.* By. E. Klein. London: McMillan & Co.

*Conférences Thèrapeutiques et Cliniquès sur les Maladies des Enfants.* Par le Dr. Jules Simon. Tome II. Paris.

*A Pharmacopoeia for the treatment of Diseases of the Larynx, Pharynx and Nasal Passages.* G. M. Lefferts, A. M., M. D. Second Edition. G. P. Putnam's Sons.

*A Manual of Bandaging.* By C. Henri Leonard, A. M., M. D. Second Edition. Illustrated Medical Journal Co., Detroit.

*The Basic Pathology and Specific Treatment of Diphtheria aud Zymotic Diseases*, by Geo. G. Ziegler, M. D.

2cM

SUBSCRIPTION PRICE, $3.00 PER YEAR.

Whole Number, 303. MARCH, 1885. Vol. L., Number 3.

LIBRARY
INDEXED
S.G.O.

### THE CHICAGO MEDICAL JOURNAL AND EXAMINER

EDITORS:

S. J. JONES, M.D., LL.D.

W. W. JAGGARD, A.M., M.D. HAROLD N. MOYER, M.D.

COLLABORATORS.

CHICAGO:

PROFESSOR J. A. ALLEN, M. D., LL. D.

PROFESSOR E. ANDREWS, M. D., LL. D.

PROFESSOR W. H. BYFORD, A. M., M. D.

PROFESSOR O. C. DEWOLF, A. M., M. D.

PROFESSOR E. C. DUDLEY, A. B., M. D.

PROFESSOR J. H. ETHERIDGE, A. M., M. D.

PROFESSOR C. FENGER, M. D.

PROFESSOR J. N. HYDE, A. M., M. D.

PROFESSOR H. A. JOHNSON, M. D., LL. D.

C. G. SMITH, A. M., M.D.

J. F. TODD, M. D.

MILWAUKEE, WISCONSIN:
N. SENN, M. D.

WASHINGTON, D. C:

S. O. RICHEY, M. D.

UNITED STATES NAVY:
MEDICAL INSPECTOR J. M. BROWN, M. D.

